# Significant Sensitivity Improvement for Camera-Based Lateral Flow Immunoassay Readers

**DOI:** 10.3390/s18114026

**Published:** 2018-11-19

**Authors:** Lalita Saisin, Ratthasart Amarit, Armote Somboonkaew, Oraprapai Gajanandana, Orawan Himananto, Boonsong Sutapun

**Affiliations:** 1 School of Electronic Engineering, Institute of Engineering, Suranaree University of Technology, 111 University Ave., Muang, Nakhon Ratchasima 30000, Thailand; gulalita_zcg@hotmail.com; 2 Photonics Technology Laboratory, National Electronics and Computer Technology Center, 112 Thailand Science Park, Phahon Yothin Rd., Pathumthai 12120, Thailand; ratthasart.amarit@nectec.or.th (R.A.); Armote.Somboonkaew@nectec.or.th (A.S.); 3 National Center for Genetic Engineering and Biotechnology, 113 Thailand Science Park, Phahon Yothin Rd., Pathumthai 12120, Thailand; oraprapai@biotec.or.th (O.G.); orawanh@biotec.or.th (O.H.)

**Keywords:** lateral flow immunoassays, LFA reader, strip reader, smartphone reader, test kits

## Abstract

Recent developments in smartphone-based strip readers have further improved the performances of lateral flow test kits. Most smartphone cameras encode an unaltered and nonlinear power-law transfer function that maps the light intensity to a pixel value; this poses some limitations for camera-based strip readers. For faint-color test lines which are almost as white such as with nitrocellulose pads, the slope of the transfer function is low. Therefore, it is difficult to differentiate between the faint test lines and the white background. We show that by manually setting the camera exposure time—instead of using the automatic settings—to the high-slope region of the transfer function, the reader’s sensitivity can be improved. We found that the sensitivity and the limit of detection of the *Acidovorax avenae* subsp. *citrulli* (*Aac*) test kit were enhanced up to 3-fold and 5-fold, respectively, when using the readers at the optimal camera settings, compared to the automatic mode settings. This simple technique can be readily applied to any existing camera-based colorimetric strip reader to significantly improve its performance.

## 1. Introduction

Rapid test kits using lateral flow immunoassays (LFA) have been widely used for point-of-care diagnostics, food analysis, and environment chemical contaminant monitoring, especially in the areas of resource-limited settings [1,2,3]. With the help of a reading device, LFAs can provide semi-quantitative results, improved repeatability, simple network connectivity, and error reduction caused by varying human visual abilities.

The rapid growth of wireless telecommunication networks and billions of subscribed smartphone users provide a remarkable opportunity for smartphone-based diagnostics. Users can use a smartphone to acquire an image of the test kits, process the image, display and transfer the results, and organize the data through the internet. In recent years, several smartphone-based LFA readers were developed using the phone’s CMOS camera to capture the LFA image and employ an external or internal light source (either in reflection or transmission modes), and an external housing attachment for minimizing light intensity variations [4,5,6,7,8,9,10,11,12]. After the LFA image is acquired by the smartphone camera, the raw red, green, and blue (RGB) image is converted into a gray-scale image. Appropriate image processing techniques are then applied to the gray-scale image to extract the locations of the control and test zones. Finally, the calibration curve is established by plotting the integrated intensity or the intensity change at the test line. 

Modern digital camera software encodes a nonlinear power-law transfer function, known as gamma (γ), that maps the input light intensity to the electrical signal output (pixel value). An unaltered γ = 0.45–0.55 is normally set by smartphone manufacturers to compensate for the nonlinear brightness response of the displays to achieve proper visual observation [13,14]. This image gamma poses limitations for camera-based LFA readers. For faint-color test lines which are almost as white as the nitrocellulose pads, the slope of the transfer function is low. Therefore, it is difficult to differentiate between faint-color test lines and the white nitrocellulose background. Since the effect of the gamma on the LFA reader’s performances has not been reported yet, we assume that previous reports employ a default gamma value.

In this work, we show that by decreasing the camera exposure time to the point with the highest slope of the camera’s transfer function, we can improve the reader sensitivity and the limit of detection of the *Aac* test kits using camera-based readers up to 3-fold and 5-fold, respectively. Using our approach, the performances of existing smartphone colorimetric LFA readers or other camera-based readers can be significantly improved.

## 2. Materials and Methods

**Figure 1:**
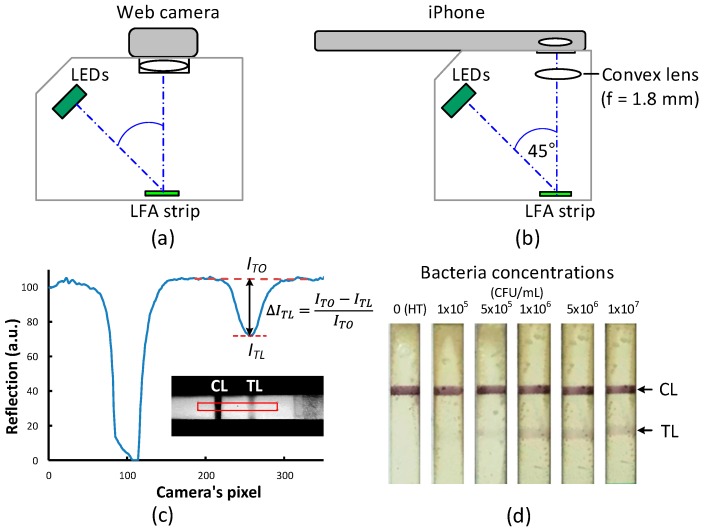
Schematic diagrams of the lateral flow readers using a web camera (**a**) and an iPhone (**b**) as a detection device. (**c**) Reflection line profile obtained from a LFA strip showing the intensity drops (Δ*I*_TL_) at the control line (CL) and the test line (TL). (**d**) Color images of LFA strips after tested with various bacteria concentrations.

Two versions of lateral flow readers were built using a consumer-grade web camera (C525, Logitech, Lausanne, Switzerland) and a smart phone camera (iPhone 5S, Apple, Cupertino, CA, USA), respectively (Figure 1). The web camera can be focused on the close range of a LFA strip without an additional lens. The iPhone, however, needs a convex lens (f = 1.8 mm) to be placed in front of the camera’s own lens in order to increase the image magnification and focus on a close range of the LFA strip. The light source for both readers is a green light emitting diode line array arranged parallel to the longitudinal axis of the test strip and at a 45° angle of incidence. All optical components were enclosed in a 3D-printed housing to block out light from the surroundings. The strip images acquired by both the web camera and the iPhone were transferred to a computer and processed by a custom program written in the LabVIEW 2014 software (National Instruments, Austin, TX, USA). To match the wavelength of the light source, only the green pixel values of the camera were extracted for further processing.

To account for non-uniform light intensities and dark signal noise, a reference image and a dark image were recorded prior to each test. The reference image was an image of a fresh LFA strip with the light source turned on, while the dark image was an image of the strip with the light source turned off. The reflection from the test strip at the *x*th camera pixel is defined as *I*_R_(*x*) = [*I*(*x*) − *I*_dark_(*x*)]/[*I*_ref_(*x*) − *I*_dark_(*x*)], where *I*(*x*), *I*_ref_(*x*), and *I*_dark_(*x*) are the intensity values recorded by the *x*th pixel from the testing strip image, the reference strip image, and the dark image, respectively. The reflection line profile of the test strip along the *x*-axis was plotted and the reflected intensity drop at the center position was calculated using ∆*I*_TL_ = (*I*_TO_ − *I*_TL_)/*I*_TO_, where *I*_TO_ is the white background brightness value determined from the fitted line, and *I*_TL_ is the brightness value at the center of the test line (Figure 1c), both calculated using our custom software.

The web camera driver directly allows an adjustment of the camera’s settings, including exposure time, gain, contrast, white balance, and brightness, except the γ value. To set all camera attributes, a used LFA strip with a clear control line and a clear test line was first placed inside the reader and the auto mode was selected to take a picture of the LFA. Next, all other camera settings were fixed except the exposure time. We then used the web camera reader to record the images of the strips tested with bacteria concentrations ranging from 0 (healthy sample) to 1 × 10^7^ CFU/mL (See Supplementary Information for more details) for the exposure times 15 ms, 31 ms, 62 ms, 125 ms (auto-mode exposure time), and 250 ms.

Adjusting the iPhone camera settings required a third-party mobile application (the Procam app.). Similarly to the web camera reader, we first inserted a used LFA strip into the iPhone reader and used the Procam app. to adjust the camera settings until both the control line and the test line were clearly observed. We then manually fixed all camera settings except the exposure time. The exposure time of the iPhone reader was varied: 8 ms, 13 ms, 17 ms, 22 ms (auto-mode exposure time), and 67 ms.

To estimate the limit of detection (LoD), we used the method proposed by Armbruster and Pry [15] in which LoD = LoB + 1.645 (SD_low sample concentration_), where LoB is the limit of a blank defined as LoB = mean_Blank_ + 1.645 (SD_Blank_), and SD is the standard deviation. Statistical differences of the presented data were analyzed using the *t*-test, considering *p* < 0.05 as statistically significant.

## 3. Results and Discussion

**Figure 2:**
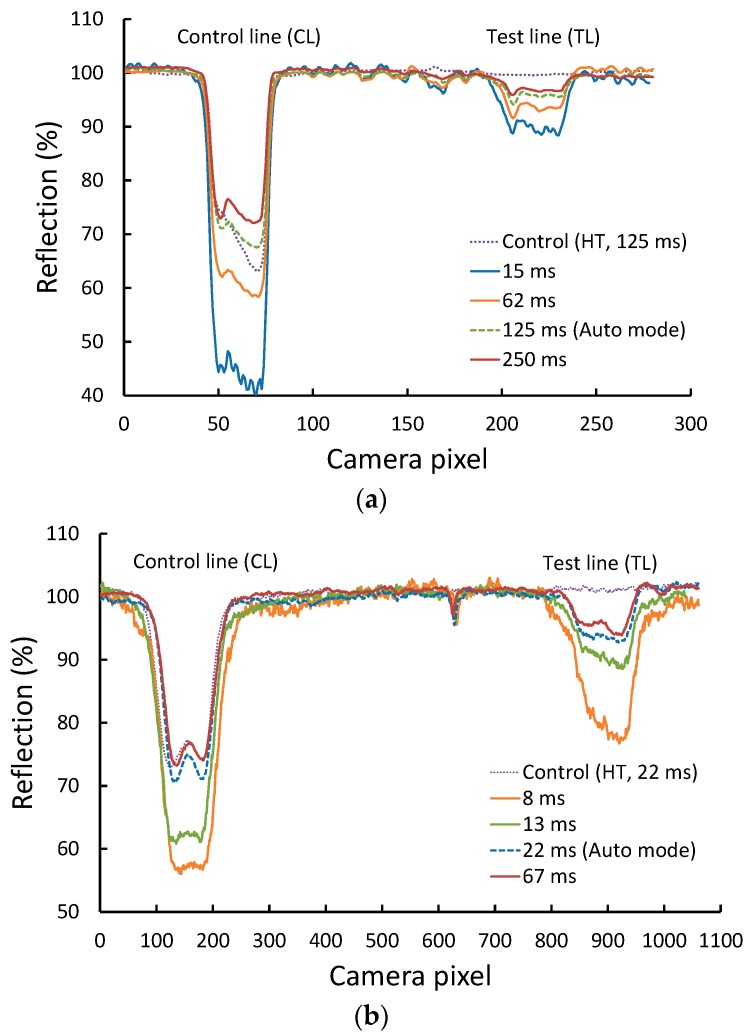
Reflected light profiles from the test area of the test strip at a bacteria concentration of 1 × 10^6^ CFU/mL. The image of the LFA strip was captured with different camera exposure times when using (**a**) a web cam reader and (**b**) an iPhone reader.

For the web camera reader, we tested the LFA strips with *Aac* concentrations of 0–1 × 10^7^ CFU/mL spiked in healthy plant sap extract. The healthy plant sap extract was used as negative control. Limit of detection for visual readout of the test kit was 1 × 10^6^ CFU/mL (Figure 1d). Figure 2a shows the reflection line profiles obtained from the strip’s test area tested with 1 × 10^6^ CFU/mL concentration with the camera exposure times of 15 ms, 61 ms, 125 ms (auto mode), and 250 ms. Note that the data for each concentration was obtained from the same strip but with its image captured successively with different exposure times. The results show that Δ*I*_TL_ significantly increases when the camera exposure time decreases. The highest Δ*I*_TL_ was obtained for the 15-ms exposure time, the shortest exposure time used in our experiment. The results obtained from the iPhone reader are shown in Figure 2b, exhibiting a similar trend as the web camera reader. The highest Δ*I*_TL_ was found with the 8-ms exposure time (see Supplementary Information for additional results). Note that the camera noise of both readers increases as the exposure time decreases and the strip images appear darker. Therefore, the camera exposure time cannot be decreased further. For both readers, Δ*I*_TL_ at the shortest exposure time is improved about 3-fold relative to the auto-mode setting (*p* < 0.05).

**Figure 3:**
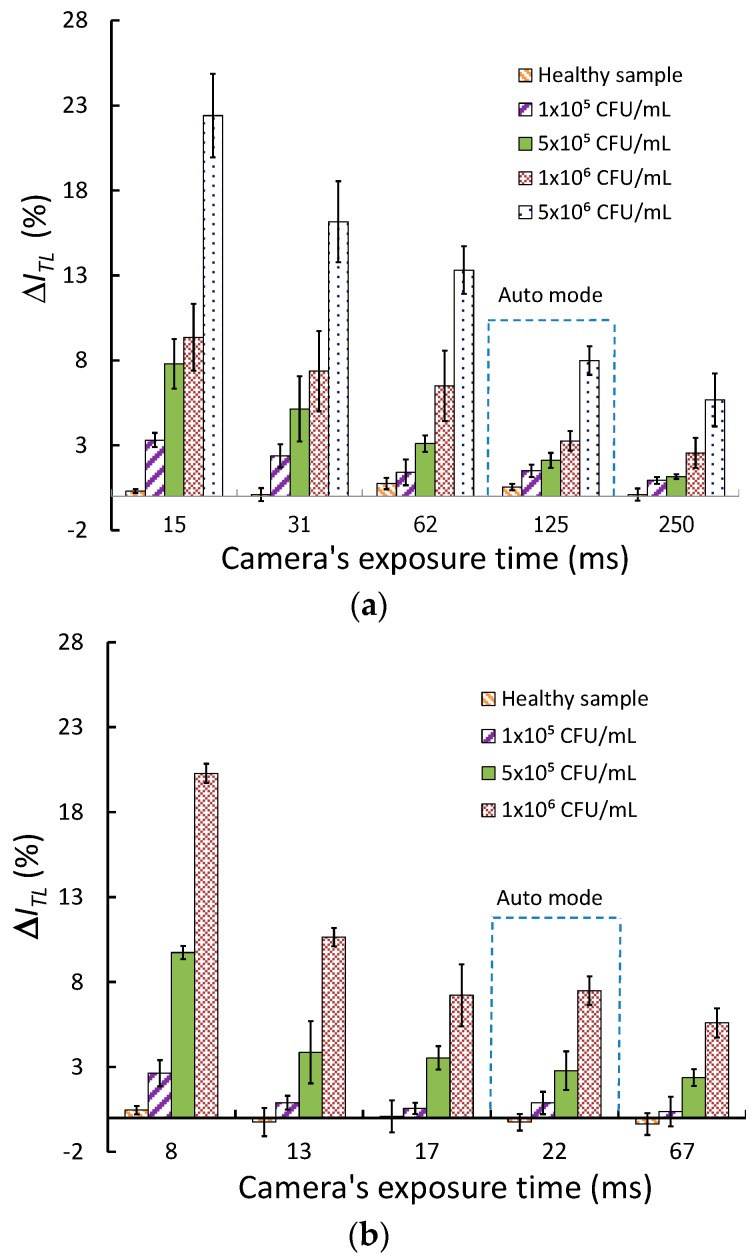
Relative intensity drops Δ*I*_TL_ at the strip’s test line for various *Aac* bacteria concentrations plotted as a function of camera exposure time for (**a**) the web-camera reader, *N* = 5 for each concentration, and (**b**) the iPhone reader, *N* = 3 for each concentration.

Figure 3a,b (see also Figure S12) show the plots of Δ*I*_TL_ for the different exposure times for various *Aac* bacteria concentrations for the web-camera reader and the iPhone reader, respectively. The results show that the reader’s sensitivity increases when the exposure time decreases for all bacteria concentrations. 

Figure S13 shows reflected line profiles obtained from the LFA’s test area with 0–1 × 10^7^ CFU/mL bacteria concentrations for 3 measurements using the webcam reader using the exposure time of 15 ms. Note that the experiments in Figure S13 were performed about one month apart from the experiments in Figure 3a using the same reader. The measured line profiles and the sensor response for each concentration were nearly identical indicating that the LFA test has a good repeatability and reproducibility. 

The calculated LoD for the *Aac* test kits is at 1 × 10^5^ CFU/mL for both the web camera reader (15-ms exposure time) and the iPhone reader (8-ms exposure time) which is 5-fold better than using the automatic settings. Compared to visual readout having an LoD of 1 × 10^6^ CFU/mL, using the readers provides a 10 folds improvement in LoD.

**Figure 4:**
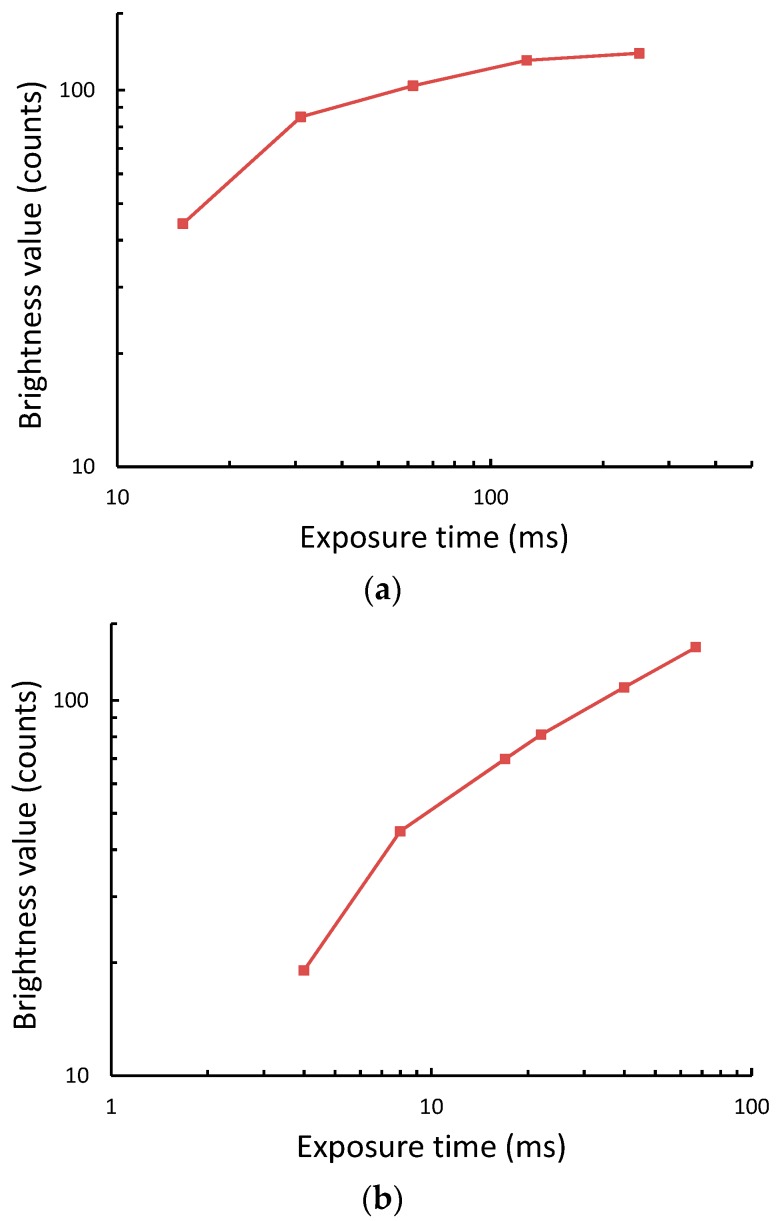
Brightness values obtained from a white background of the test strip as a function of camera exposure time (**a**) using the web camera reader and (**b**) using the iPhone reader.

To confirm the CMOS cameras have non-linear transfer functions, we measured the image brightness value of the white background area of the test strip as a function of camera exposure time. We found that the brightness values for both cameras decrease in a nonlinear fashion, as expected, exhibiting a higher slope at the lower range of the exposure time (Figure 4). Nonetheless, both cameras have different transfer functions since they have different camera settings, including gain, contrast, brightness, and white balance.

It has been known in the field of image processing that a higher slope of the transfer function corresponds to higher the image contrast [13]. The Weber’s image contrast of the test line defined as (*I_TL_* − *I*_BG_)/*I*_BG_, where *I*_BG_ is the brightness value of the white background, is similar to the reader sensitivity, Δ*I*_TL_, presented in this work. Therefore, we can conclude that reducing the camera exposure time enhances the image contrast of the test line resulting in a larger difference in pixel values between the test line and the white nitrocellulose pad.

This approach, however, may not be applied to chemiluminescence or fluorescence lateral flow systems since the signal intensities of both systems are quite low compared to the signal intensity of a colorimetric LFA. For low brightness images, the camera’s transfer function is already in a high-slope region. Setting the camera exposure time to a shorter value in this case will not improve the reader performance. In fact, it will rather deteriorate the reader performance because the signal intensity as well the signal-to-noise ratio will decrease significantly.

Preechaburana et al. [12] evaluated high-dynamic-range (HDR) processing to improve the image contrast of lateral flow strips. In their work, three strip images were successfully recorded with exposure values (EV) of 0 EV (auto mode), +2 EV (overexposure by 2 stops) and −2 EV (underexposure by 2 stops) and then converted to an HDR image using a specialized software. The device sensitivity was improved approximately 2-fold using the HDR processing. However, the HDR processing involves tone mapping, which is an intensive computational task. Another disadvantage of this technique is that the camera exposure time must change to three different values for every test, which is usually not permitted by most smartphone operating systems. Compared to the HDR processing, our technique does not demand extensive computation, and it is a simple operation since the camera exposure time is fixed for all tests.

For cameras or smartphone cameras that allow gamma adjustment, γ = 1 (linear response) should be set and the exposure time should be increased, not decreased as proposed in this work, to achieve maximum signal-to-noise ratio.

## 4. Conclusions

For camera-based LFA readers–including smart phone readers–that normally employ an unaltered nonlinear power-law gamma curve, using automatic camera settings is not an optimal condition for the detection of a faint-color test line of an LFA strip. The image of the white nitrocellulose background and the faint-color test line have a high brightness value and the camera software maps this input light intensity onto an output signal with in the low-slope region of the transfer function. To improve the device sensitivity, the camera exposure time should be manually set to lower values than set by the camera’s automatic mode, in which the slope of the transfer function is the highest and the signal-to-noise ratio is still sufficiently large. We found that by using the optimal exposure time, the device sensitivity and the limit of detection of camera-based readers for the *Aac* test kit increase about 3-fold and up to 5-fold, respectively, compared to those obtained using the automatic mode. This simple but effective technique could be employed to significantly improve the sensitivity and the detection limit of smartphone and other camera-based colorimetric LFA readers without hardware changes.

## Supplementary Materials

The following are available online at http://www.mdpi.com/1424-8220/18/11/4026/s1, Figure S1: Schematic illustration of the LFA strip for *Aac* detection. TL is sprayed with MAb 11E5 (Test line) and CL is sprayed with goat anti-mouse IgG (Control line). Conjugate pad is sprayed with colloidal gold-MAb 11E5 conjugate. Figure S2: Result interpretation for application of Aac-LFA strip. Figure S3: Grayscale images of the LFA strip and corresponding reflected light profiles from the test area of the test strip at a bacteria concentration of 5 × 10^6^ CFU/mL. Note that the data was obtained from the same strip but with its image captured successively with different exposure times: (a) 250 ms, (b) 125 ms, (c) 62 ms, (d) 31 ms and (e) 15 ms. For low exposure time, the LFA images appear dark and difficult to see by naked eye. Figure S4: Grayscale images of the LFA strip and corresponding reflected light profiles from the test area of the test strip at a bacteria concentration of 1 × 10^6^ CFU/mL. Note that the data was obtained from the same strip but with its image captured successively with different exposure times: (a) 250 ms, (b) 125 ms, (c) 62 ms, (d) 31 ms and (e) 15 ms. For low exposure time, the LFA images appear dark and difficult to see by naked eye. Figure S5: Grayscale images of the LFA strip and corresponding reflected light profiles from the test area of the test strip at a bacteria concentration of 5 × 10^5^ CFU/mL. Note that the data was obtained from the same strip but with its image captured successively with different exposure times: (a) 250 ms, (b) 125 ms, (c) 62 ms, (d) 31 ms and (e) 15 ms. For low exposure time, the LFA images appear dark and difficult to see by naked eye. Figure S6: Grayscale images of the LFA strip and corresponding reflected light profiles from the test area of the test strip at a bacteria concentration of 1 × 10^5^ CFU/mL. Note that the data was obtained from the same strip but with its image captured successively with different exposure times: (a) 250 ms, (b) 125 ms, (c) 62 ms, (d) 31 ms and (e) 15 ms. For low exposure time, the LFA images appear dark and difficult to see by naked eye. Figure S7: Grayscale images of the LFA strip and corresponding reflected light profiles from the test area of the test strip at a bacteria concentration of 0 CFU/mL (healthy sample). Note that the data was obtained from the same strip but with its image captured successively with different exposure times: (a) 250 ms, (b) 125 ms, (c) 62 ms, (d) 31 ms and (e) 15 ms. Figure S8: Grayscale images of the LFA strip and corresponding reflected light profiles from the test area of the test strip at a bacteria concentration of 1 × 10^6^ CFU/mL. Note that the data was obtained from the same strip but with its image captured successively with different exposure times: (a) 67 ms, (b) 22 ms, (c) 17 ms, (d) 13 ms and (e) 8 ms. For low exposure time, the LFA images appear dark and difficult to see by naked eye. Figure S9: Grayscale images of the LFA strip and corresponding reflected light profiles from the test area of the test strip at a bacteria concentration of 5 × 10^5^ CFU/mL. Note that the data was obtained from the same strip but with its image captured successively with different exposure times: (a) 67 ms, (b) 22 ms, (c) 17 ms, (d) 13 ms and (e) 8 ms. For low exposure time, the LFA images appear dark and difficult to see by naked eye. Figure S10: Grayscale images of the LFA strip and corresponding reflected light profiles from the test area of the test strip at a bacteria concentration of 1 × 10^5^ CFU/mL. Note that the data was obtained from the same strip but with its image captured successively with different exposure times: (a) 67 ms, (b) 22 ms, (c) 17 ms, (d) 13 ms and (e) 8 ms. For low exposure time, the LFA images appear dark and difficult to see by naked eye. Figure S11: Grayscale images of the LFA strip and corresponding reflected light profiles from the test area of the test strip at a bacteria concentration of 0 CFU/mL (healthy sample). Note that the data was obtained from the same strip but with its image captured successively with different exposure times: (a) 67 ms, (b) 22 ms, (c) 17 ms, (d) 13 ms and (e) 8 ms. For low exposure time, the LFA images appear dark and difficult to see by naked eye. Figure S12: The normalized reflected intensity drop at the center position of the test line (∆*I*_TL_) plotted as a function of bacteria concentrations of spiked samples for the webcam reader (a) (N = 5) and the iPhone reader (b) (N = 3), respectively. Figure S13: The reflection line profiles obtained from the strip’s test area tested with 0−1 × 10^7^ CFU/mL concentrations with the camera exposure time of 15 ms using the web-camera reader. The experiments were repeated 3 times.
