# Supplementary material for: Significant Sensitivity Improvement for Camera-Based Lateral Flow Immunoassay Readers
Source: Sensors (Basel). 2018 Nov 19;18(11):4026. doi: 10.3390/s18114026 (PMC6263405; doi:10.3390/s18114026)
Supplement: Supplementary file 1 [file sensors-18-04026-s001.pdf]

Supplementary Information

Significant sensitivity improvement for camera-based lateral flow immunoassay readers

Lalita Saisin [1], Ratthasart Amarit [2], Armote Somboonkaew [2], Oraprapai Gajanandana 3, Orawan Himananto [3], and Boonsong Sutapun[1,*]

[1] School of Electronic Engineering, Institute of Engineering, Suranaree University of Technology, 111 University Ave., Muang, Nakhon Ratchasima 30000, Thailand
[2] Photonics Technology Laboratory, National Electronics and Computer Technology Center, 112 Thailand Science Park, Phahon Yothin Rd., Pathumthai 12120, Thailand
[3] National Center for Genetic Engineering and Biotechnology, 113 Thailand Science Park, Phahon Yothin Rd., Pathumthai 12120, Thailand
* Correspondence: boonsong@sut.ac.th

Received: date; Accepted: date; Published: date

S1. Reagents and Materials

Mouse monoclonal antibody specific to *Acidovorax avenae* subsp. *citrulli; Aac* (MAb 11E5) was produced by the Monoclonal Antibody Production Laboratory, National Center for Genetic Engineering and Biotechnology, Thailand [1]. Bovine serum albumin (BSA), sodium borate decahydrate ($Na_2B_4O_7$), Goat anti-mouse IgG (M 8642) were purchased from Sigma. Colloidal gold (40-nm diameter), glass fiber, polyester sheet, adsorbent pad, nitrocellulose membrane and plastic backing were obtained from Pacific Biotech Co. Ltd., Thailand.

S2. Fabrication of Lateral Flow Devices

S2.1 Preparation of the colloidal gold-MAb conjugate

To prepare the colloidal gold-MAb conjugate, purified MAb 11E5 was resuspended in phosphate buffer (PB) pH 7.4 to get the final concentration of 1 mg/ml. Colloidal gold (0.2 ml) was added to the antibody solution (2 ml) and gently mixed on the magnetic stirrer at room temperature. After incubation for 1 h, 10% BSA in PB (20 ml) was added and stirred at 200 rpm for another 1 h. The mixture was centrifuged at 9,000 rpm for 40 min, and the pellet was resuspended in 1 ml of 0.02 M borate buffer containing 5% trehalose and 20% sucrose, and stored at 4 °C in dark until use.

S2.2 Preparation of lateral flow immunoassay strip

The LFA strip for detection of *Aac* was developed as previously described using mouse monoclonal antibody (MAb 11E5) specific to *Aac* [2]. Briefly, the purified MAb 11E5 was conjugated with colloidal gold particles as described above and sprayed onto a glass fiber (conjugate pad) at 2 µl/cm. The same MAb (1 mg/ml) was sprayed onto a nitrocellulose membrane at 1 µl/cm and used as the capture antibody at the test line. Goat anti-mouse IgG (1 mg/ml) was sprayed onto the same nitrocellulose membrane at 1 µl/cm and used as the capture antibody at the control line. After drying the conjugate pad and nitrocellulose membrane for 2 h at 37 °C, the components of the strips, i.e. sample pad, conjugate pad, nitrocellulose membrane and absorbent pad were assembled on plastic backing (Figure S1). The assembled strip test was cut into 3.5 mm-wide strips and stored in a self-sealing plastic bag with desiccant at room temperature.

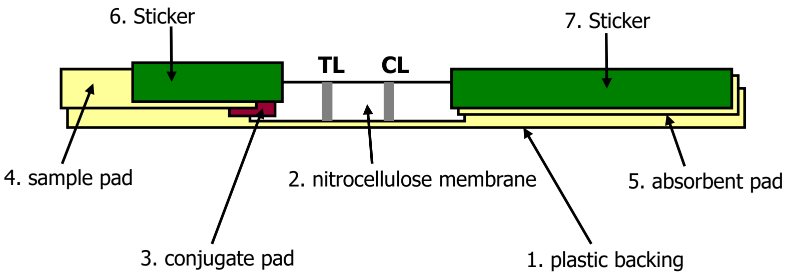

Figure S1 Schematic illustration of the LFA strip for Aac detection. TL is sprayed with MAb 11E5 (Test line) and CL is sprayed with goat anti-mouse IgG (Control line). Conjugate pad is sprayed with colloidal gold-MAb 11E5 conjugate.

Positive result for *Aac* showed reddish purple bands on the test (TL) and control (CL) lines whereas negative result showed only one reddish purple band on the control line (CL). If no band was observed on the control line, the results were deemed invalid and were discarded (Figure S2).

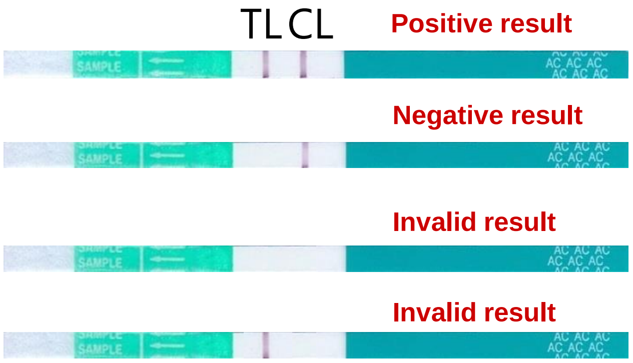

Figure S2 Result interpretation for application of Aac-LFA strip.

S3. Samples and Spiked Sample Preparation

S3.1 Acidovorax avenae subsp. *citrulli* (*Aac*)

Aac strain KK9 used in this study was previously isolated from infected watermelon as described by Himananto et al. [1]. To prepare the bacterial cell suspensions, *Aac* was cultured on Nutrient agar and incubated at 28°C for 48 h. A single colony was selected and inoculated into 50 ml of Nutrient broth. The cultures were incubated at 28°C for 18 h, with shaking at 200 rpm. The bacteria were harvested by centrifugation at 5,000 rpm for 10 min. The cell pellets were washed twice with 0.1 M phosphate buffered saline (PBS) and resuspended in the same buffer to obtain a final concentration of 1×10^{10} CFU/ml.

S3.2 Sample Preparation

S3.2.1 *Aac*-infected and healthy samples

Watermelon leaves were ground in the extraction buffer ($Na_2B_4O_7$ buffer, pH 8.6) at a 1:5 w/v ratio. After 1-min incubation at room temperature, the plant sap was transferred to a clean tube for further tests.

S3.2.2 *Aac*-spiked samples

Plant extract from healthy leaves was prepared as described above. Varying concentrations of *Aac* (0-10^7 CFU/ml) were added to the healthy plant sap before LFA strip analysis.

S3.2.3 Application of LFA strips

Insert the LFA strip into the sample tube containing approximately 0.25 ml of plant sap. The results could be observed within 5 min after sample application. Result interpretation is shown in Figure S2.

S4. Results from the Web-Camera Reader

Figure S3-S7 show original grayscale images of the LFA strips and the reflection line profiles obtained from the strip's test area tested with 0–5×10^6 CFU/mL concentrations with the camera exposure times of 15 ms, 61 ms, 125 ms (auto mode), and 250 ms. Note that the images and data for each concentration were obtained from the same strip but with its image captured successively with different exposure times. The images of the LFA strips with low exposure time values were relatively dark and were difficult to observe visually unless some contrast enhancement techniques were applied. Nonetheless, the reflection line profiles of these LFA strips showed a clear intensity drop at the test line when the strips tested with 1×10^5–5×10^6 CFU/mL concentrations.

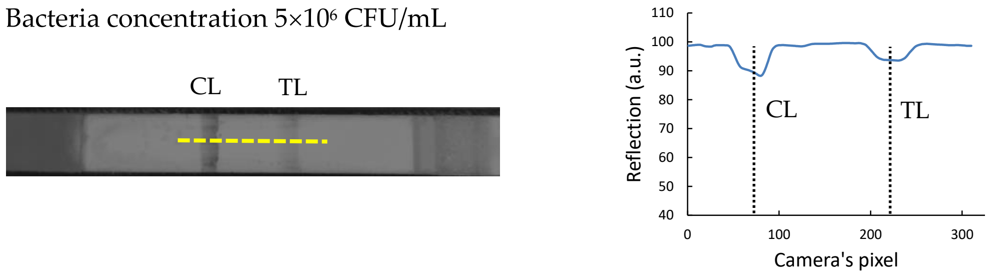

(a) Exposure time = 250 ms

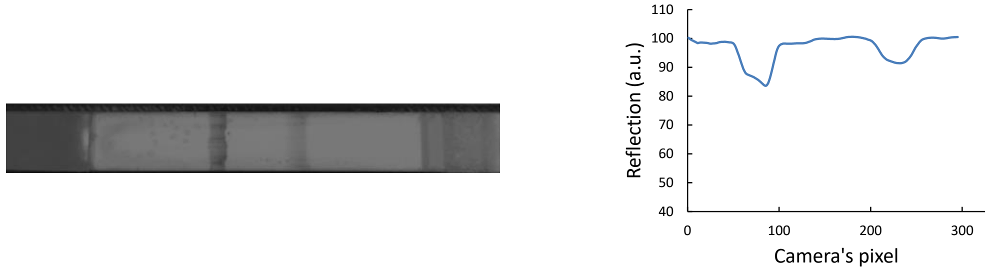

(b) Exposure time = 125 ms

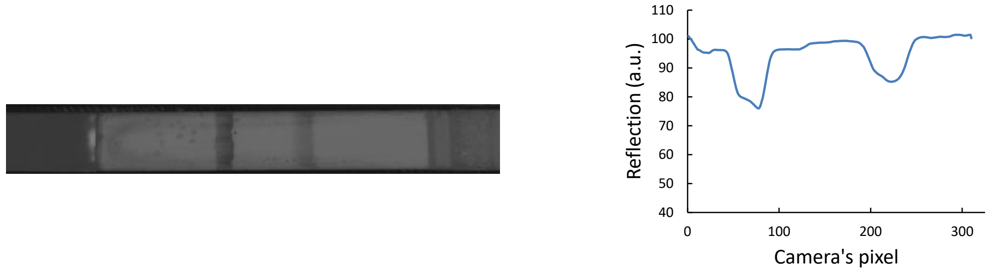

(c) Exposure time = 62 ms

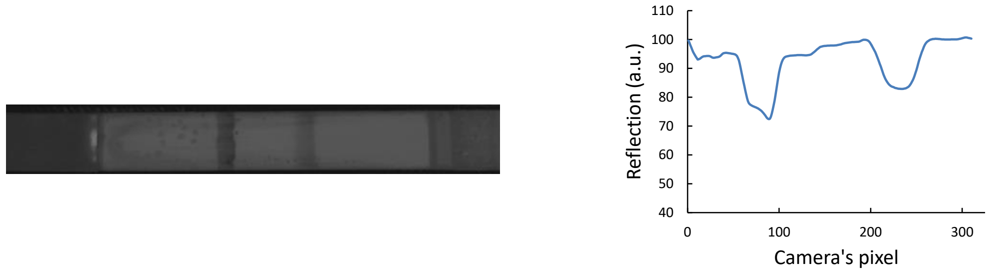

(d) Exposure time = 31 ms

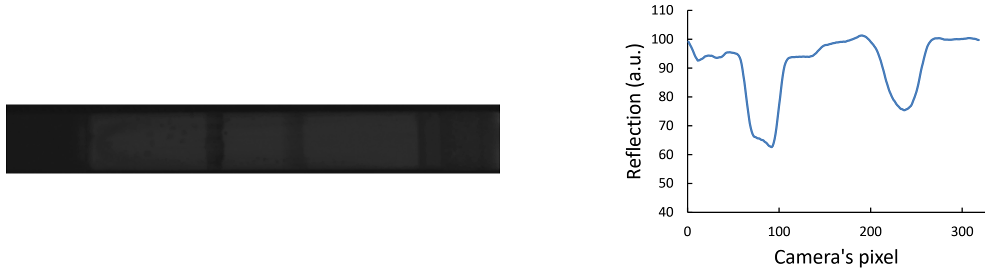

(e) Exposure time = 15 ms

Figure S3 Grayscale images of the LFA strip and corresponding reflected light profiles from the test area of the test strip at a bacteria concentration of 5×10^6 CFU/mL. Note that the data was obtained from the same strip but with its image captured successively with different exposure times: (a) 250 ms, (b) 125 ms, (c) 62 ms, (d) 31 ms and (e) 15 ms. For low exposure time, the LFA images appear dark and difficult to see by naked eye.

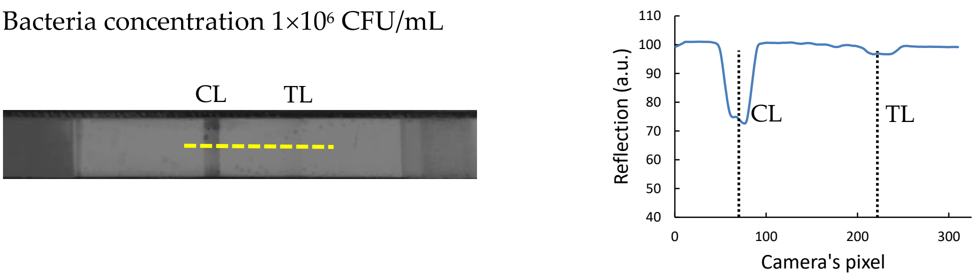

(a) Exposure time = 250 ms

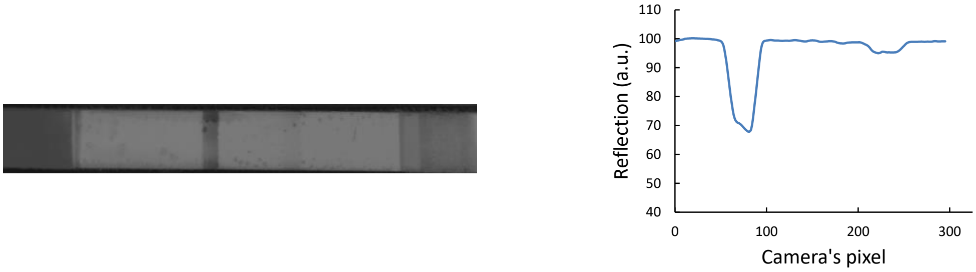

(b) Exposure time = 125 ms

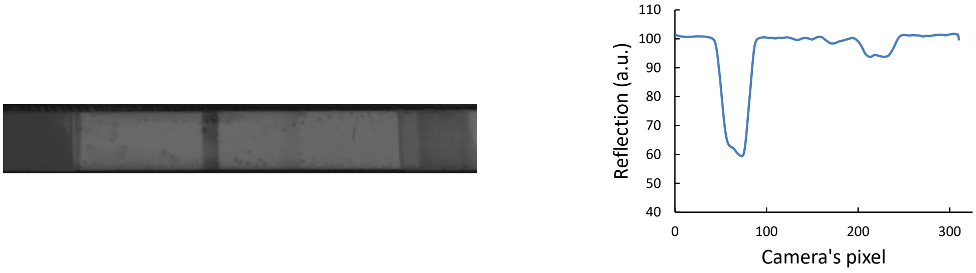

(c) Exposure time = 62 ms

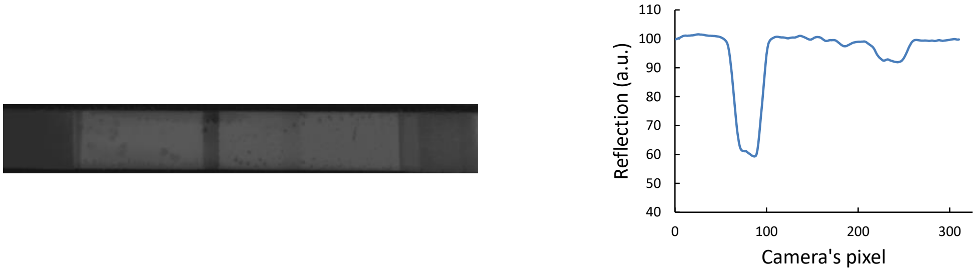

(d) Exposure time = 31 ms

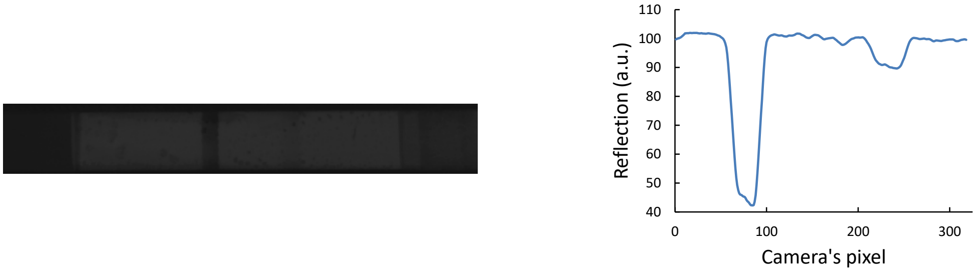

(e) Exposure time = 15 ms

Figure S4 Grayscale images of the LFA strip and corresponding reflected light profiles from the test area of the test strip at a bacteria concentration of 1×10^6 CFU/mL. Note that the data was obtained from the same strip but with its image captured successively with different exposure times: (a) 250 ms, (b) 125 ms, (c) 62 ms, (d) 31 ms and (e) 15 ms. For low exposure time, the LFA images appear dark and difficult to see by naked eye.

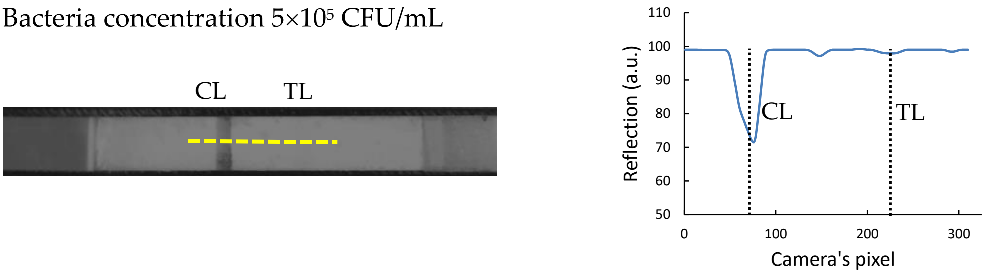

(a) Exposure time = 250 ms

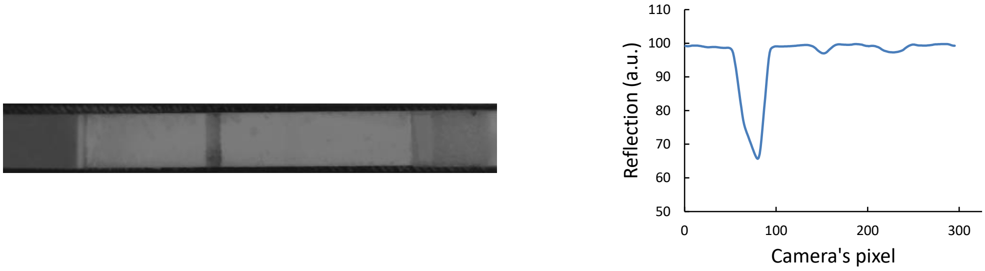

(b) Exposure time = 125 ms

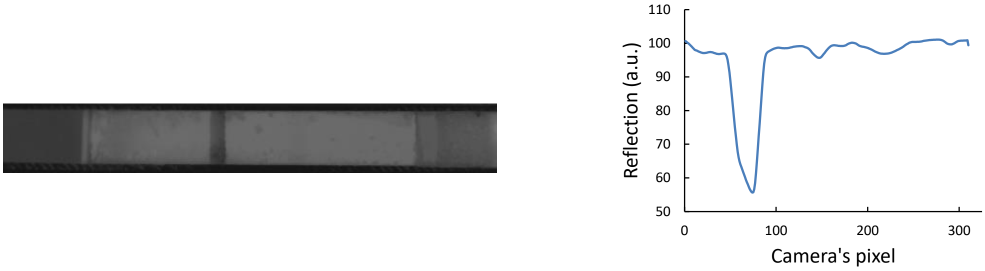

(c) Exposure time = 62 ms

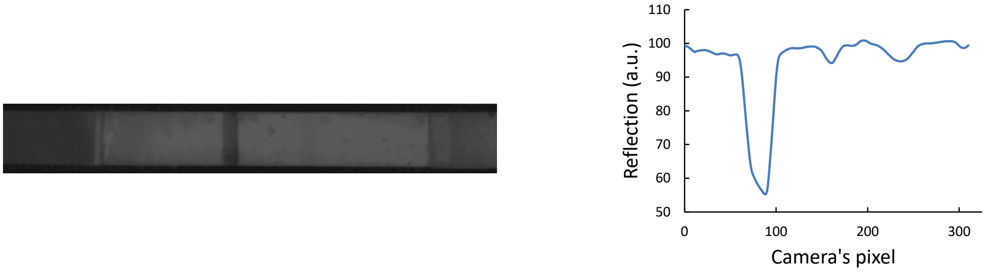

(d) Exposure time = 31 ms

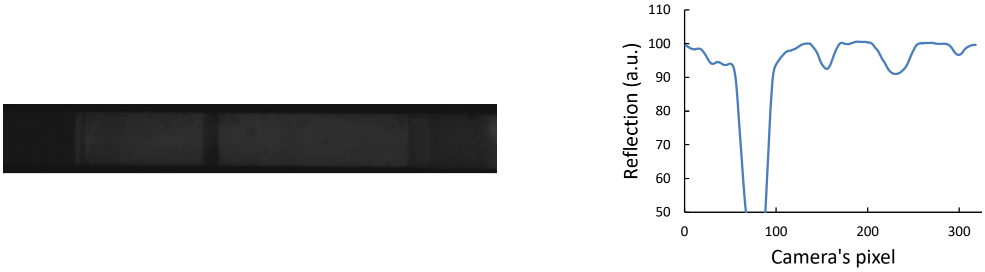

(e) Exposure time = 15 ms

Figure S5 Grayscale images of the LFA strip and corresponding reflected light profiles from the test area of the test strip at a bacteria concentration of 5×10^5 CFU/mL. Note that the data was obtained from the same strip but with its image captured successively with different exposure times: (a) 250 ms, (b) 125 ms, (c) 62 ms, (d) 31 ms and (e) 15 ms. For low exposure time, the LFA images appear dark and difficult to see by naked eye.

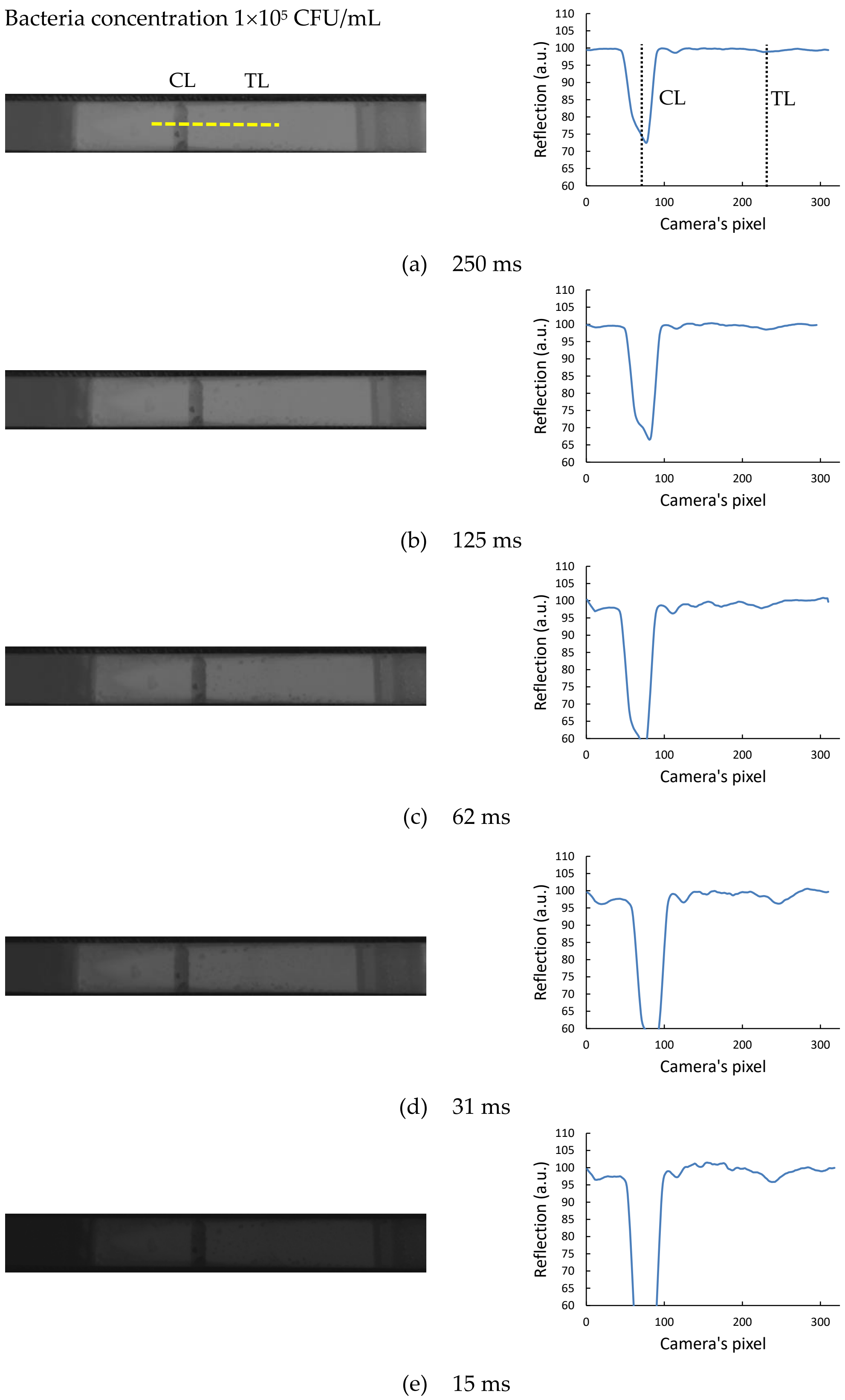

Figure S6 Grayscale images of the LFA strip and corresponding reflected light profiles from the test area of the test strip at a bacteria concentration of 1×10^5 CFU/mL. Note that the data was obtained from the same strip but with its image captured successively with different exposure times: (a) 250 ms, (b) 125 ms, (c) 62 ms, (d) 31 ms and (e) 15 ms. For low exposure time, the LFA images appear dark and difficult to see by naked eye.

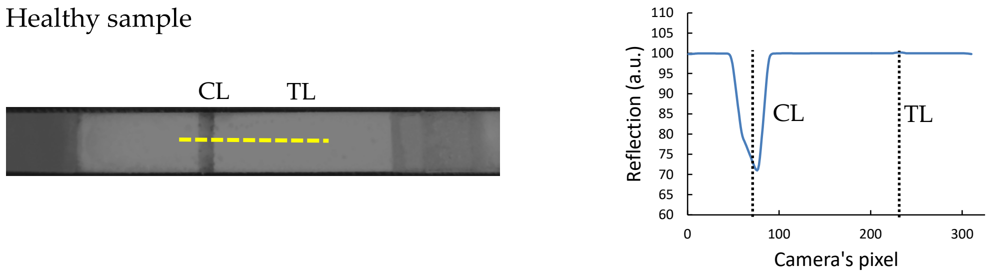

(a) 250 ms

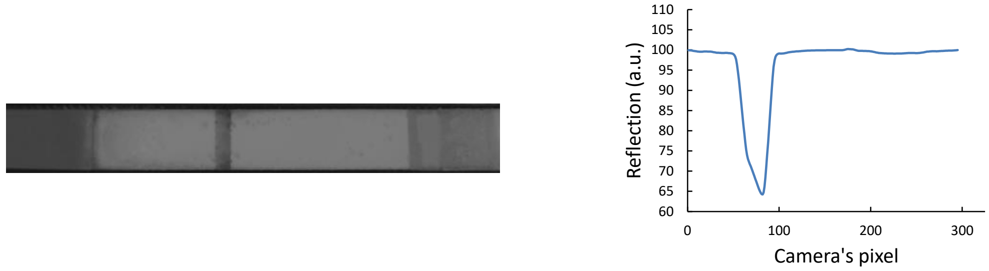

(b) 125 ms

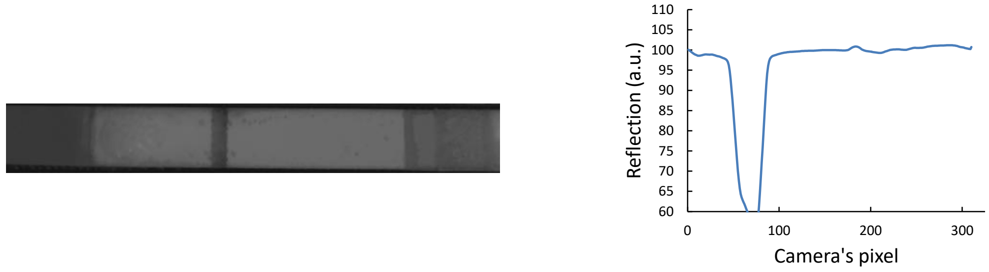

(c) 62 ms

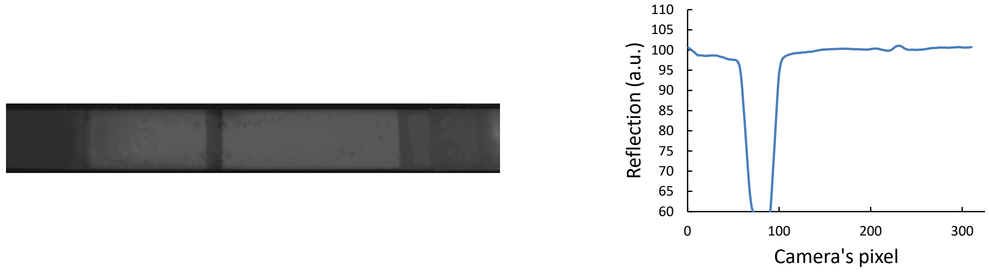

(d) 31 ms

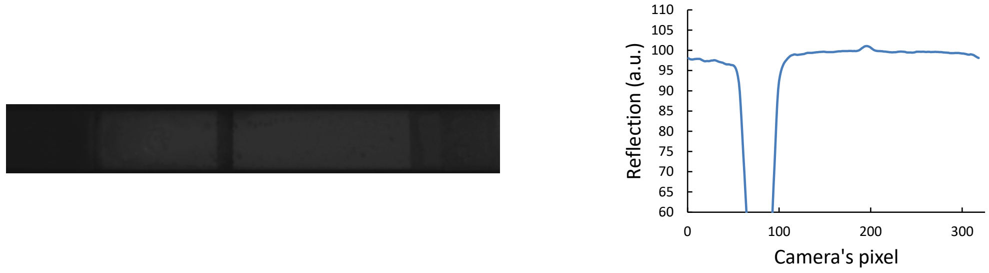

(e) 15 ms

Figure S7 Grayscale images of the LFA strip and corresponding reflected light profiles from the test area of the test strip at a bacteria concentration of 0 CFU/mL (healthy sample). Note that the data was obtained from the same strip but with its image captured successively with different exposure times: (a) 250 ms, (b) 125 ms, (c) 62 ms, (d) 31 ms and (e) 15 ms.

S5. Results from the Mobile-Phone Reader

Figure S8-S11 show the original grayscale images of the LFA strips and the reflection line profiles obtained from the strip's test area tested with 0–1×10^6 CFU/mL concentrations with the camera exposure times of 8 ms, 13 ms, 17 ms, 22 ms (auto mode), and 67 ms. Note that the images and data for each concentration were obtained from the same strip but with its image captured successively with different exposure times. The images of the LFA strips with low exposure time values were relatively dark and were difficult to observe visually unless some contrast enhancement techniques were applied. Nonetheless, the reflection line profiles of these LFA strips showed a clear intensity drop at the test line when the strips tested with 1×10^5–1×10^6 CFU/mL concentrations.

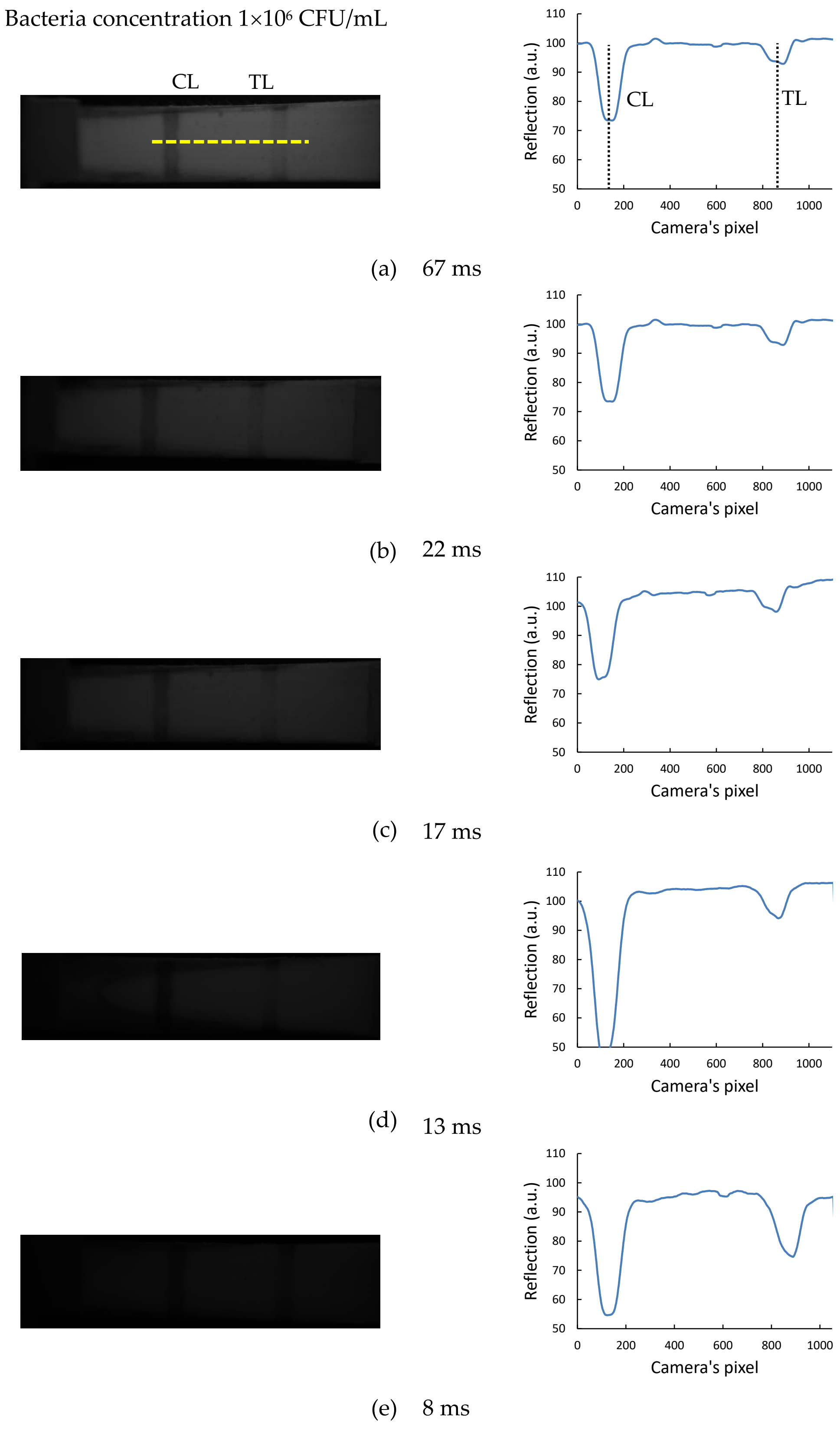

Figure S8 Grayscale images of the LFA strip and corresponding reflected light profiles from the test area of the test strip at a bacteria concentration of 1×10^6 CFU/mL. Note that the data was obtained from the same strip but with its image captured successively with different exposure times: (a) 67 ms, (b) 22 ms, (c) 17 ms, (d) 13 ms and (e) 8 ms. For low exposure time, the LFA images appear dark and difficult to see by naked eye.

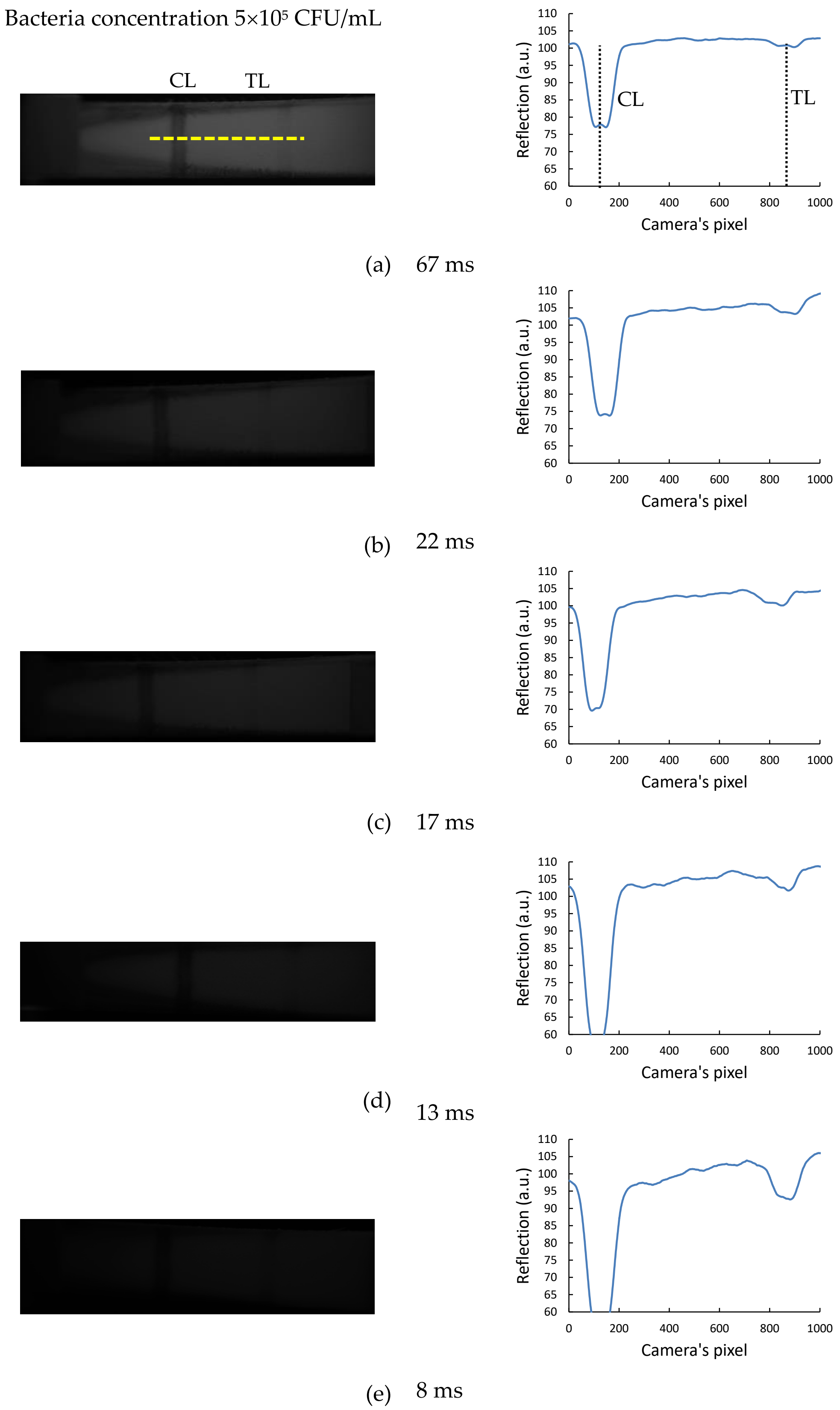

Figure S9 Grayscale images of the LFA strip and corresponding reflected light profiles from the test area of the test strip at a bacteria concentration of 5×10^5 CFU/mL. Note that the data was obtained from the same strip but with its image captured successively with different exposure times: (a) 67 ms, (b) 22 ms, (c) 17 ms, (d) 13 ms and (e) 8 ms. For low exposure time, the LFA images appear dark and difficult to see by naked eye.

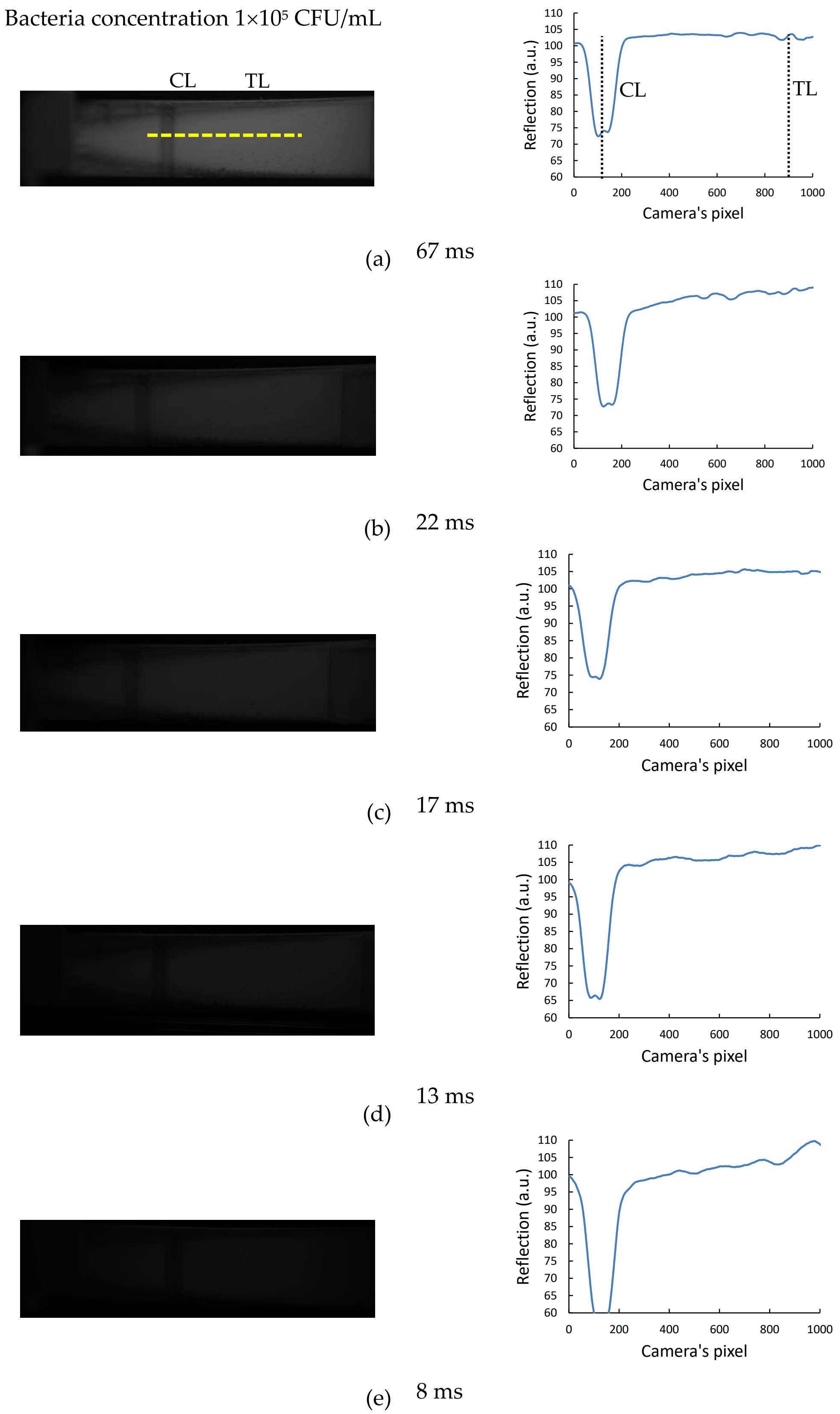

Figure S10 Grayscale images of the LFA strip and corresponding reflected light profiles from the test area of the test strip at a bacteria concentration of 1×10^5 CFU/mL. Note that the data was obtained from the same strip but with its image captured successively with different exposure times: (a) 67 ms, (b) 22 ms, (c) 17 ms, (d) 13 ms and (e) 8 ms. For low exposure time, the LFA images appear dark and difficult to see by naked eye.

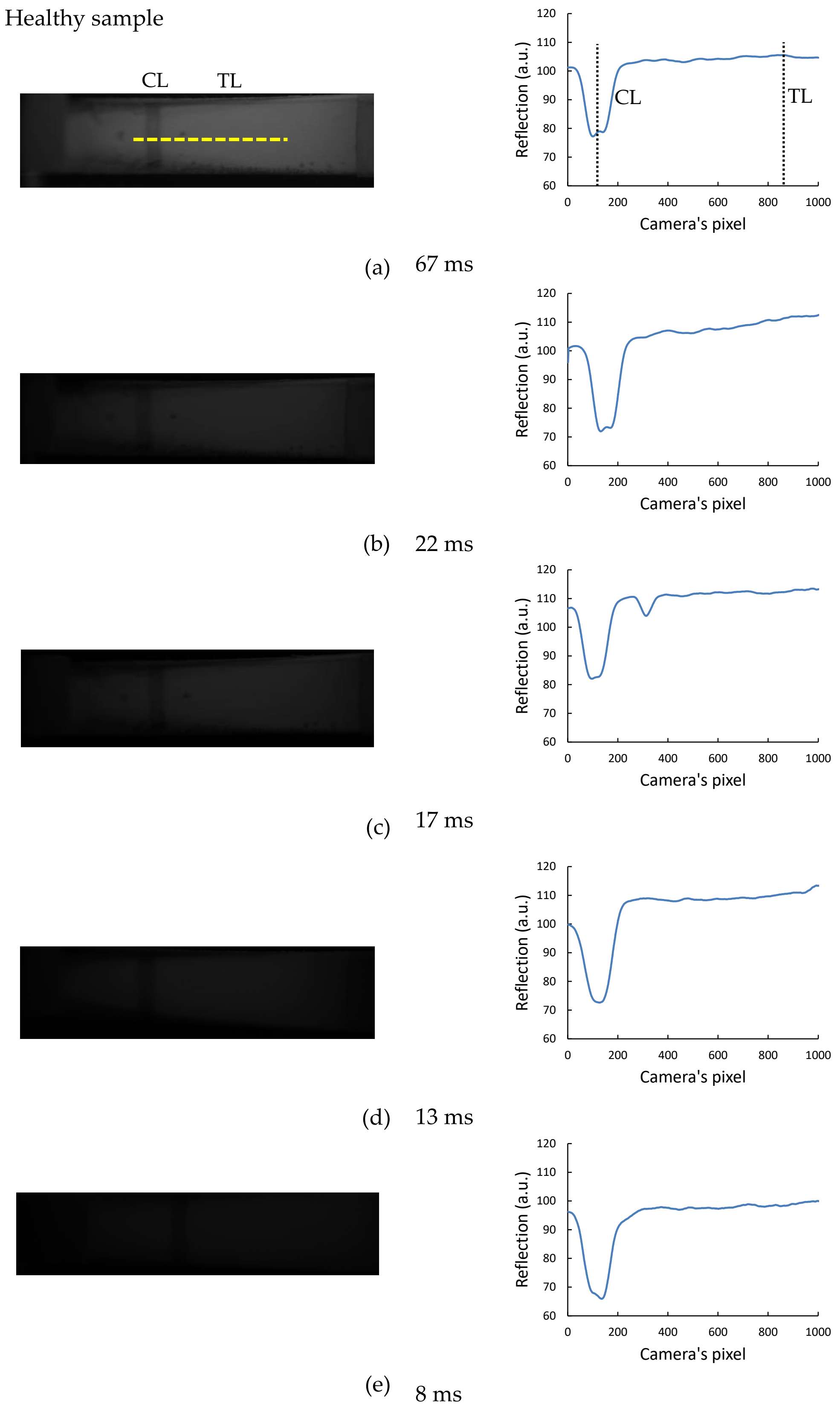

Figure S11 Grayscale images of the LFA strip and corresponding reflected light profiles from the test area of the test strip at a bacteria concentration of 0 CFU/mL (healthy sample). Note that the data was obtained from the same strip but with its image captured successively with different exposure times: (a) 67 ms, (b) 22 ms, (c) 17 ms, (d) 13 ms and (e) 8 ms. For low exposure time, the LFA images appear dark and difficult to see by naked eye.

S6. Sensitivity and Reproducibility

Figure S12 shows the reflected intensity drop at the center position of the test line (ΔI_{TL}) as a function of bacteria concentrations for the webcam reader and the iPhone reader, respectively. The data in this figure are the same data as in Figure 3 but with ΔI_{TL} plotted as a function of bacteria concentrations for two different exposure times, the shortest exposure time and the auto exposure time, for each reader. The results confirm that for both readers using the shortest exposure time provides a higher sensitivity than using the auto exposure time.

Figure S13 shows reflected line profiles obtained from the LFA's test area with 0–1×10^7 CFU/mL bacteria concentrations for 3 measurements using the webcam reader. The exposure time of the web-camera reader was set at 15 ms. The results were nearly identical indicating that the LFA test has a good short-term repeatability. The experiments in Figure S13 were performed about one month apart from the experiments in Figure S12 using the same reader. At 1×10^5 CFU/mL, ΔI_{TL} = 3.46±0.43% (N = 3) and 3.31±0.42% (N = 5) and when using negative control (healthy sample), ΔI_{TL} = 0.22±0.004% (N = 3) and 0.30±0.12% (N = 5), for both experiments, respectively. The LFA test results using the proposed readers show a good reproducibility. Nonetheless more data are required to support a statistically significant conclusion.

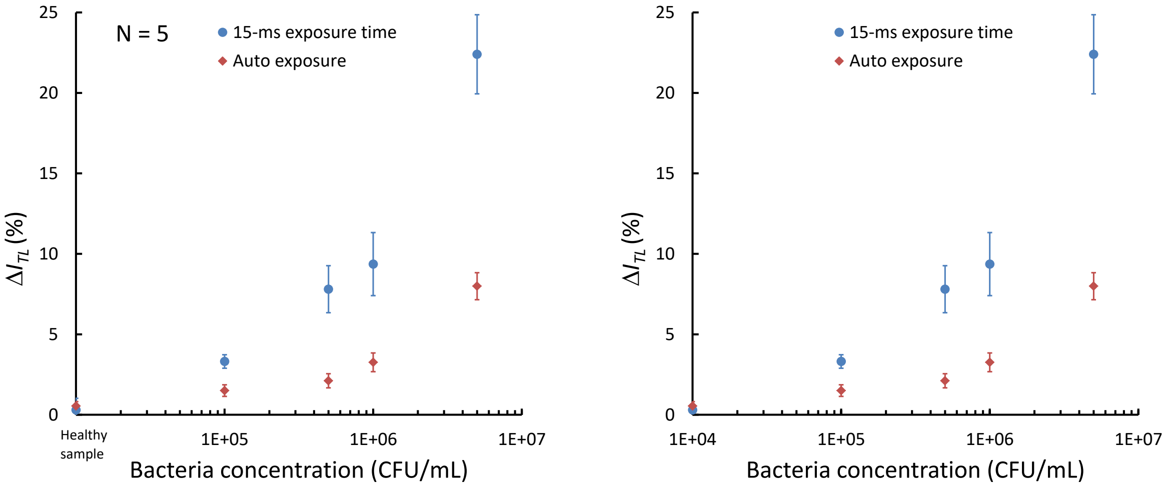

(a) (b)

Figure S12 The normalized reflected intensity drop at the center position of the test line (ΔI_{TL}) plotted as a function of bacteria concentrations of spiked samples for the webcam reader (a) (N = 5) and the iPhone reader (b) (N = 3), respectively.

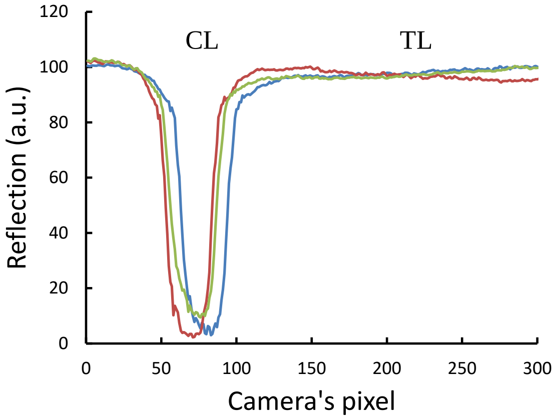

(a) Healthy sample (0 CFU/mL)

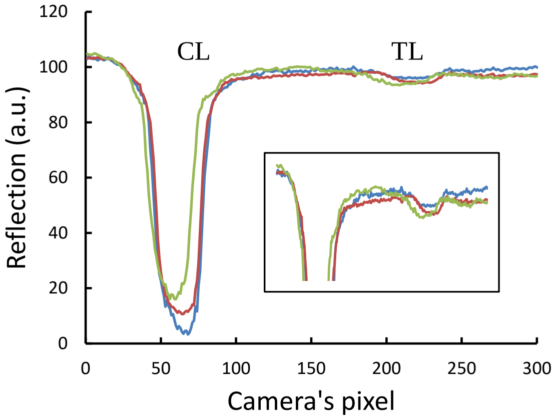

(b) 1×10[5] CFU/mL

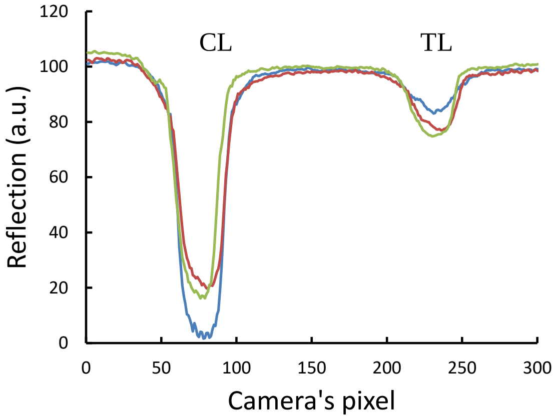

(c) 5×10[5] CFU/mL

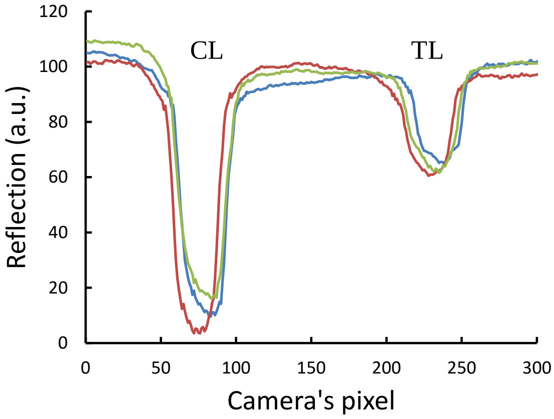

(d) 1×10[6] CFU/mL

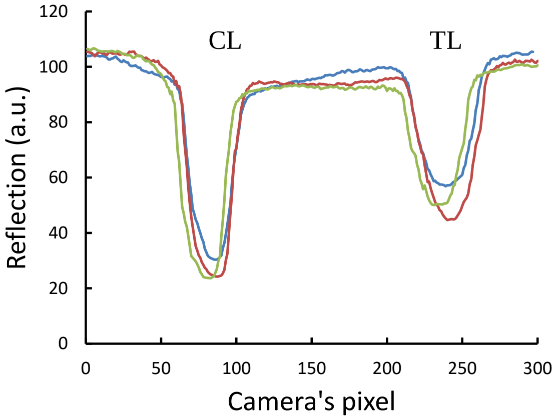

(e) 5×10[6] CFU/mL

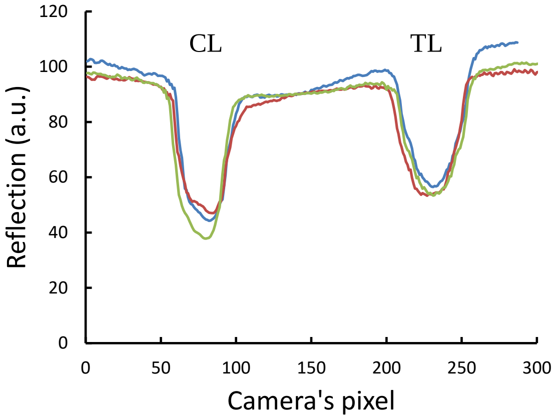

(f) 1×10[7] CFU/mL

Figure S13 The reflection line profiles obtained from the strip's test area tested with 0–1×10[7] CFU/mL concentrations with the camera exposure time of 15 ms using the web-camera reader. The experiments were repeated 3 times.

References

1. Himananto, O.; Thummabenjapone, P.; Luxananil, P.; Kumpoosiri, M.; Hongprayoon, R.; Kositratana, W.; Gajanandana, O. Novel and highly specific monoclonal antibody to *Acidovorax citrulli* and development of ELISA-based detection in cucurbit leaves and seeds. *Plant Disease* **2011**, *95*, 1172-1178.
2. Gajanandana, O.; Himananto, O.; Kumpoosiri, M.; Luxananil, P.; Thummabenjapone, P.; Kiratiya-angul, S. Development of immunochromatographic strip test for the rapid detection of *Acidovorax avenae* subsp.

citrulli in plant samples. ABIC 2009: Agricultural Biotechnology International Conference, Queen Sirikit National Convention Center, Bangkok, Thailand, 22-25 September 2009, P183

© 2018 by the authors. Submitted for possible open access publication under the terms and conditions of the Creative Commons Attribution (CC BY) license (http://creativecommons.org/licenses/by/4.0/).
